# A Speech Delivered on Behalf of Ancoats Hospital
*An address given to the delegates of the Manchester and Salford Hospital Saturday Fund at Onward Buildings, Deansgate, on May 8, 1914, by Mr. Herbert J. Dafforne, Secretary Ancoats Hospital, Manchester.


**Published:** 1914-05-30

**Authors:** 


### THE ART OF APPEALING TO WORKING MEN.

### A Speech Delivered on Behalf of Ancoats Hospital.*

Hospital secretaries in the course of their work deliver a large number of speeches which seldom, if ever, survive beyond the hour of their delivery. As no two hospitals and no two audiences are the same we have thought it well, in the interests of mutual co-operation, to print an abstract of one such speech recently made by Mr. H. J. Dafforne, secretary of Ancoats Hospital, Manchester. It is at once a history, an appeal, and a revelation to working men of hospital work Other secretaries who make speeches of the kind, into which much research and hard work have gone, are invited to send them to us, so that new ideas for hospital appeals and old experience in presenting facts to special audiences, which at present are individual traits and nothing more, may become the common property of institutional workers through the pages of The Hospital.

---

It is not my intention to weary you with statistics, but simply to give a short history of the growth and needs of Ancoats Hospital, Manchester, and to try so to infuse the spirit of hospital life into each of you that you may, in your turn, enthuse others in the great cause of hospitals. Ancoats Hospital was first launched forth in the reign of George IV., when even your great city of Manchester was not allowed a representative in Parliament and when the first railway between Manchester and Liverpool was being constructed. On August 11, 1828, Ancoats Hospital was started as a public dispensary for the district of Ardwick and Ancoats. One of the rules is especially of interest, and has caused our present doctors much amusement. It reads: "That no amputation or other great operation be performed without a previous consultation of a physician and the surgeons of the institution." One wonders what would happen if this rule was still adhered to.

### The Art of Appeal in 1845.

The institution for a number of years was only open for the treatment of home patients and out-patients. Of the way in which the gentlemen in the past appealed for funds here is an example from the annual report of 1845:—

"Everyone knows that a neglected feverish cold may end in typhus fever, communicable to thousands, an influenza in consumption, a rheumatic fever in incurable heart-disease, a kick or a bruise in permanent lameness if neglected, sore eyes in blindness, and so on. Now," the report adds, "if those so affected were not in a condition to obtain medical assistance when it might have availed them, it is evident that they will be unable to maintain themselves when incapacitated for labour, and that this expense must fall on the public. When the poor man is sick all must be sacrificed to the hope of regaining that health and strength which is his only capital." One large item of expenditure during the first twenty-five years of the dispensary's existence was for leeches, for which a sum of £25 to £50 was paid annually.

In 1866 the committee rejoiced for the first time since the foundation of the dispensary that it was out of debt. In 1872 strenuous and successful efforts were being made towards raising funds for the purchase of a new site.

In 1873 Miss Brackenbury gave £5,000, which the trustees of the dispensary spent in erecting a noble hospital with every accommodation for surgical and dispensing practice on land in Mill Street. Three spacious wards were set aside for hospital patients, and it was hoped that funds would soon be raised to enable the committee to supply fifty beds. Miss Brackenbury left by her will a further sum of £2,000. The formal opening of the new buildings took place on January 29, 1874, by the then Bishop of Manchester (Dr. Fraser). The Bishop went on to remark that he was at first inclined to doubt whether Mill Street, Great Ancoats, was altogether the most salubrious spot in Manchester in which to place a hospital, but he noticed that during the previous year 1,994 patients were cases of accidents which would be much more effectively treated in the wards of a hospital than in the houses of the people themselves.

In 1874, forty years ago, the present chairman of the hospital, Mr. W. Armitage, joined the committee of management, and it is a wonderful record of personal service which Mr. Armitage has given to the sick poor of Ancoats.

### The Condition of Ancoats in 1884.

The following extract from the report of the Medical Officer of Health for the City of Manchester was included in the annual report of the hospital for the year 1884.

> Ancoats is a district requiring, I think, distinct and special consideration. Though a part of the old township of Manchester, it is really a somewhat outlying portion of the city, and has received but scant justice. It is a compact block of cottage houses, most of them of the meanest character, housing some 48,000 of the most industrious people in the world. It covers an area of 360 acres, and has in portions a very dense population. The oldest outlying district of Manchester, it must for many years have contributed largely by its mills and manufactories to the rates of the town and city. With the population of a considerable town, grouped as no equal population in any part of the kingdom probably is grouped, it has not a single thoroughfare traversing its length. With a population outside it equal to that of a large city, it has not a single road or street enabling that vast population to communicate in a fairly straight line with the city with which its business chiefly lies.

In 1886 Mr. James Jardine offered to endow a ward in the hospital containing twelve beds with £500 per annum, and a capital sum of £12,500 was given by him to yield this amount in perpetuity. The committee had at this time thirty-seven beds for patients, and urgently appealed for more accommodation and improved sanitary arrangements, also an additional ward for women, and better sleeping apartments for the nurses. The cost was estimated at about £5,000.

October 20, 1888, must be marked as a red-letter day in the hospital's career, as it was on this day that the late Prince Albert Victor of Wales laid the foundation-stone of the wing that by special permission is called the Albert Victor Pavilion. The President of the hospital

---

* An address given to the delegates of the Manchester and Salford Hospital Saturday Fund at Onward Buildings, Deansgate, on May 8, 1914, by Mr. Herbert J. Dafforne, Secretary Ancoats Hospital, Manchester.

was Mr. J. Jardine. The additions made to the hospital at this time comprised six new wards capable of accommodating eighty-four patients, four special wards of one bed each, a complete steam laundry for the officials and patients, a mortuary and *post-mortem* and disinfecting chamber, together with the official department, nurses' and servants' sleeping and day rooms.

The first mention as to the desirability of having a convalescent home connected with the hospital was in 1890. To quote from the annual report, the appeal reads: "The priceless boon of a few weeks' residence in the pure air of a country house to a weak and pale-faced patient is an absolute necessity. A gift of £10,000 would provide such a home. Who will send our Treasurer a cheque for this amount?" Ancoats district must have been a place of pure air and country houses in the memory of one of the speakers at the annual meeting of 1891, for he says: "In the old days they used to think Ancoats was the Land of Goschen, and the fine-cotton spinners then considered themselves the aristocracy."

Through the exertions of the ladies' committee a convalescent home for women only was secured at Wilmslow in 1891. In 1894 the committee recorded their sense of the loss to the institution caused by the death of their President, Mr. James Jardine. The Jardine Ward, with its fourteen beds, is to-day a living memorial of his work. Sir W. H. Holdsworth was appointed as the new President in 1895, and still holds that position.

In this year the committee had to record the death of their Treasurer, Mr. James Oliver, and in 1897 the members of his family undertook to provide an out-patient and accident room department to his memory.

This department has been in constant use since it was opened in 1900. In 1900 the trustees of the late Mr. Frederick Rothwell endowed a ward in the hospital by the payment of £10,000. In 1900 an anonymous donor offered £10,000 for the building of a convalescent home, and in July 1904 the new convalescent home was opened at Sandlebridge, Great Warford, Alderley Edge, and commenced its work.

In 1907 an *x*-ray apparatus was presented to the institution by Mr. and Miss Hyde, and a new mortuary and *post-mortem* room was built, while much-needed improvements were made in the operating theatre. In 1908 additional bedrooms for nurses had to be built, and the old hand lift was altered to an electrically driven one through the kindness of Mr. Erskine Crossley, who defrayed the cost. In the year 1909, through the removal of the Royal Infirmary from Piccadilly, the committee had to face a great demand on their resources for the treatment of accident cases, the increase in six months of that year being 2,831 over the corresponding six months of the previous year.

I will now give you the figures of the patients treated and the operations performed during 1903 and 1913, to show the increased growth, and therefore its increased usefulness.

| | 1903 | 1913 | | |
|---|---|---|---|---|
| Home patients ... | ... 2.036 | ... 697 | ... decrease | 1,339 |
| Out-patients ... | ... 5 894 | ... 8,873 | ... increase | 2,979 |
| Accident cases | ... 10,764 | ... 21,898 | ... do. | 11,134 |
| In-patients ... | ... 1.285 | ... 2 115 | ... do. | 830 |
| Operations performed | 781 | ... 1.754 | ... do. | 973 |

You will notice the great increase in accident cases during these ten years; many of these cases really ought to be admitted for inside treatment, but until the public provides us with more beds we cannot do the impossible. During 1913 alone 504 of our admissions to the wards were accident cases, calling for immediate admission.

The various prices paid in 1903 and 1913 for various commodities used at the institution were as follows:

| | 1903 | 1913 | |
|---|---|---|---|
| Bread per 4lb. loaf ... | -/3¾ ... | -/4¼ | £ 18 per an. |
| Meat per lb. ... ... | -/6 ... | -/7¼ | 103 ,, |
| Milk, per doz. pts. ... | 1/11½ ... | 2/4½ | 69 ,, |
| House coal per ton ... | 13/7 ... | 18/- | 60 ,, |
| Making a total increase on these four items alone of ... ... | | | £250 per an.* |

* Estimated extra cost on the present consumption.

The first great need is for more adequate support in the shape of new and increased annual subscriptions. We are quite willing to have annual subscribers from 1s. upwards per annum; there is no need to wait until you can afford your guinea. We are quite willing to have a 5s. subscription list included in our annual report; we only await the subscribers. Last year our

| | |
|---|---|
| Annual subscriptions were ... ... | £3,336 |
| Dividends from invested funds ... | 2,123 |
| Your society gave us ... ... ... | 350 |
| Hospital Sunday ... ... ... ... | 304 |
| Donations and other unreliable income | 2,331 |
| Making a total income of ... ... | £8,444 |

Our expenditure amounted to £10,227, leaving a deficit of over £1,500 to be made up. Now surely this is not a very hard task in Manchester. In Leicester the hospital benefits to the tune of £14,000 through the systematic collection of one penny per week voluntarily given by the workers.

Finally, we need at once some £600 for sanitary equipment and roof reconstruction. We also urgently require a new accident ward and further house room for nurses at a cost of £3,000 or more. £700 a year more income is required for the convalescent home.

We leave the cause of Ancoats Hospital and its great needs with the general public; the responsibility rests with them. The committee await their mandate to provide the needs I have enumerated—will they wait in vain?

The patients do what they can. The other day a miner was so much pleased at the progress his little girl aged five was making that he insisted upon my taking 1s. for each week she was inside the hospital, which he said was his "bacca" money, and which he would willingly go without.

